# 191 Fostering Self-Sufficiency Among Elderly Life: Perspective of Functional Ability to Achieve Healthy Aging

**DOI:** 10.1093/eurpub/ckae114.169

**Published:** 2024-09-26

**Authors:** Suditi Singh, Atul Kumar Pandey

## Abstract

**Purpose:**

Elderly life refers to the post-older adult (45-59 years) phase, when individuals reach 60 years old. The Basic Activities of Daily Living (BADL) such as feeding, dressing, bathing, grooming etc. is a fundamental component to assess the healthy ageing among elderly in India. These activities associated with caregivers, nursing homes and healthcare services (Arokiasamy et al., 2021). The circumstance is compounded by additional social inequalities, including caste and class, which intersect and lead to the emergence of many kinds of marginalization encountered by the elderly population (Bloom et al., 2021). The purpose of this study is to assess the major functional ability issues among elderly to in terms of BADL/IADLs, considering socioeconomic inequalities.

**Methods:**

Research uses Longitudinal Ageing Study in India (LASI) 2017-19 having 31464 sample from age 60 years and above. The bivariate and multivariate regression methods help to examine the variations among population.

**Results:**

In Indian context, at least one BADL was performed by 26.4% of females and 20.9% of males. Furthermore, it was found that males (33.9%) and females (56.7%) necessitated support with a minimum of one Instrumental Activity of Daily Living (IADL). Approximately 25.7% of females and 23.8% of males required assistance with both BADLs and IADLs. IADL had an odds ratio of 1.69 (95% CI: 1.60-1.78, p < 0.001), whereas BADL dependence had an odds ratio of 1.35 (95% CI: 1.27-1.44, p < 0.001). In terms of IADLs, the most prevalent limitation identified was the inability to navigate unfamiliar environments; this occurred at rates of 19.3% for males and 39.1% for females. Comparing those in the highest income quintile to those in the lowest income quintile, the likelihood of requiring assistance with at least one ADL was 0.89 (95% CI: 0.82-0.98, p < 0.05).

**Conclusion:**

Socio-economic factors play a vital role in determining the dependence for BADL and IADL among elderly. In Indian context elderly and long-term care help to enhance the impact of universal health coverage under various age and healthcare preference cohort.

**Support/Funding Source:**

This study does not have any funding support or source from the institution or outside.

